# *Notes from the Field*: Rapidly Linking an Outbreak of *Salmonella* Typhimurium Infections to Domestically Grown Cantaloupes Through Early Collaboration — United States, 2022

**DOI:** 10.15585/mmwr.mm7305a5

**Published:** 2024-02-08

**Authors:** Colin Schwensohn, Benjamin Schneider, Erin Jenkins, Allison Wellman, Sharon Seelman Federman, Oluwakemi Oni, Nicole Stone, Jennifer Adams, Laura Gieraltowski

**Affiliations:** ^1^Division of Foodborne, Waterborne, and Environmental Diseases, National Center for Emerging and Zoonotic Infectious Diseases, CDC; ^2^Oak Ridge Institute for Science and Education, Oak Ridge, Tennessee; ^3^Food and Drug Administration, Silver Spring, Maryland; ^4^Iowa Department of Health and Human Services; ^5^Indiana Department of Health; ^6^Association of Public Health Laboratories, Silver Spring, Maryland.

**Summary**

**What is already known about this topic?**

A 2020 outbreak of *Salmonella *infections was found to be associated with melons after conclusion of harvesting, when melons were no longer likely to be on the market.

**What is added by this report?**

In 2022, whole genome sequencing (WGS)–based *Salmonella* surveillance, historical melon farm environmental sampling results, and patient interviews were used to rapidly link a *Salmonella* Typhimurium outbreak to contaminated cantaloupes.

**What are the implications for public health practice?**

WGS-based surveillance, combined with rapid collection of epidemiologic data by state and local agencies, can be used to reduce the time to outbreak detection and response.

In 2020, federal and state regulators conducted environmental testing at a midwestern melon farm in response to a multistate outbreak of *Salmonella* infections that was associated with melon consumption (*1*). *Salmonella* was detected in the environmental samples, and whole genome sequencing (WGS) was performed. PulseNet, CDC’s molecular subtyping network for foodborne disease surveillance, was used to assess genetic relationships between environmental *Salmonella* isolates and those from ill persons. *Salmonella* Typhimurium identified in environmental testing was related to illnesses in previous years that exhibited a seasonal pattern (Figure). Although this environmental strain was not linked to illnesses in the 2020 outbreak, the pattern of increased incidence during previous summers raised concern about the possibility of a persistent *Salmonella* reservoir with potential to cause future outbreaks. Investigations identified the short melon growing season as a challenge: by the time an outbreak is detected, epidemiologic and traceback evidence collected, and a farm identified, the growing season is over, and melons are no longer on the market. To overcome this challenge, CDC collaborated with the Food and Drug Administration (FDA) and state and local health and agricultural agencies in 2022 to identify all cases of *Salmonella* infection genetically related to the 2020 environmental strain for immediate follow-up. Ill persons were interviewed using a standardized questionnaire to identify the source of melon exposure as quickly as possible and before the end of the melon growing season. This activity was reviewed by CDC, deemed not research, and was conducted consistent with applicable federal law and CDC policy.*

**FIGURE:**
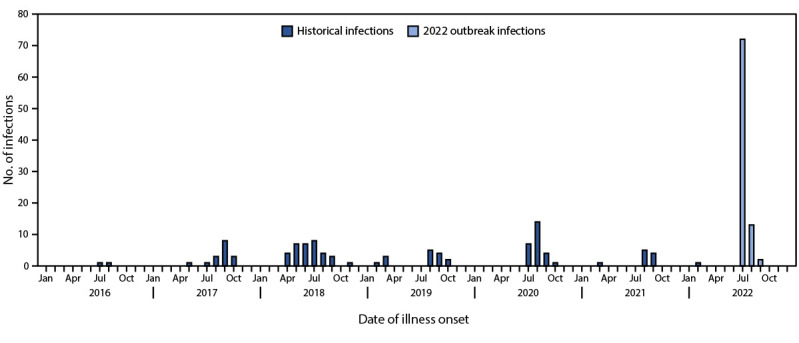
Number of persons infected with *Salmonella* Typhimurium, by case status and date of illness onset — United States, July 23, 2016–September 11, 2022

## Investigation and Outcomes

On August 4, 2022, PulseNet identified 12 *S.* Typhimurium infections that were genetically related within seven allele differences by WGS to the 2020 environmental strain. Cases were defined as infections with isolates that were related to the 2020 strain within 10 allele differences and that occurred during July 7–September 11, 2022. In total, 87 outbreak cases from 11 states were identified in 2022.^†^ The median patient age was 65 years (range = 1–93 years); 67% of patients were female. Thirty-two (37%) patients were hospitalized; none died.

Upon outbreak detection, investigators worked with state and local agencies to assess cantaloupe and watermelon exposure, which were vehicles of interest based on previous outbreak investigations. In 2022, cantaloupe consumption was reported significantly more frequently by ill persons (36 of 47; 77%) than during a 2018–2019 survey of healthy persons conducted on FoodNet sites (29%, p<0.001) (*2*). FDA traced the source of cantaloupes purchased by ill persons to a common geographic region close to where the 2020 *Salmonella* environmental strain was identified. By August 25, 2022, the combination of epidemiologic and traceback data and relationship to the 2020 environmental strain indicated that cantaloupes grown in the Midwest were the likely outbreak source. At the time cantaloupes were identified as the source, the 2022 cantaloupe growing season (May–July) had already ended (*3*). As a result, contaminated melons were unlikely to be on the market; therefore, a recall was not initiated because ongoing foodborne illness risk had ceased. In 2022, the time from outbreak detection to determining melons were the likely source was 14 days shorter compared to the 2020 outbreak investigation, which ranged from September 18, 2020–October 23, 2020. 

## Preliminary Conclusions and Actions

Although the risk for foodborne illness from contaminated melons had ended before definitive public health action could be taken, this investigation highlights how WGS-based surveillance combined with rapid epidemiologic data collection by state and local agencies can be used to reduce the time to outbreak detection and response. The time from outbreak detection to source identification was 2 weeks shorter in 2022 compared with that during the 2020 outbreak. This shortened time frame is attributable to collaboration with partners to prepare to rapidly assess food exposures after illnesses with the 2020 environmental strain were identified. In the future, these activities, paired with prospective melon sampling and *Salmonella* testing might identify melon-associated outbreak strains earlier, further speeding outbreak investigations by quickly narrowing to a likely source. The strategies detailed in this report might increase the likelihood of public health action during future outbreaks.

## Footnotes

* 45 C.F.R. part 46.102(l)(2), 21 C.F.R. part 56; 42 U.S.C. Sect. 241(d); 5 U.S.C. Sect. 552a; 44 U.S.C. Sect. 3501 et seq.

† Georgia (one case), Illinois (five), Indiana (17), Iowa (38), Kentucky (three), Michigan (three), Minnesota (four), Missouri (two), Ohio (three), South Carolina (one), and Wisconsin (10).
